# Complex problems need detailed solutions: Harnessing multiple data types to inform genetic management in the wild

**DOI:** 10.1111/eva.12715

**Published:** 2018-12-26

**Authors:** Catherine E. Grueber, Samantha Fox, Elspeth A. McLennan, Rebecca M. Gooley, David Pemberton, Carolyn J. Hogg, Katherine Belov

**Affiliations:** ^1^ Faculty of Science, School of Life and Environmental Sciences The University of Sydney Sydney New South Wales Australia; ^2^ San Diego Zoo Global San Diego California; ^3^ Save the Tasmanian Devil Program DPIPWE Hobart Tasmania Australia; ^4^ Toledo Zoo Toledo Ohio; ^5^ Zoo and Aquarium Association Australasia Mosman New South Wales Australia

**Keywords:** AlleleRetain, genetic rescue, major histocompatibility complex, microsatellites, stochastic population modelling, Tasmanian devil, translocation

## Abstract

For bottlenecked populations of threatened species, supplementation often leads to improved population metrics (genetic rescue), provided that guidelines can be followed to avoid negative outcomes. In cases where no “ideal” source populations exist, or there are other complicating factors such as prevailing disease, the benefit of supplementation becomes uncertain. Bringing multiple data and analysis types together to plan genetic management activities can help. Here, we consider three populations of Tasmanian devil, *Sarcophilus harrisii*, as candidates for genetic rescue. Since 1996, devil populations have been severely impacted by devil facial tumour disease (DFTD), causing significant population decline and fragmentation. Like many threatened species, the key threatening process for devils cannot currently be fully mitigated, so species management requires a multifaceted approach. We examined diversity of 31 putatively neutral and 11 MHC‐linked microsatellite loci of three remnant wild devil populations (one sampled at two time‐points), alongside computational diversity projections, parameterized by field data from DFTD‐present and DFTD‐absent sites. Results showed that populations had low diversity, connectivity was poor, and diversity has likely decreased over the last decade. Stochastic simulations projected further diversity losses. For a given population size, the effects of DFTD on population demography (including earlier age at death and increased female productivity) did not impact diversity retention, which was largely driven by final population size. Population sizes ≥500 (depending on the number of founders) were necessary for maintaining diversity in otherwise unmanaged populations, even if DFTD is present. Models indicated that smaller populations could maintain diversity with ongoing immigration. Taken together, our results illustrate how multiple analysis types can be combined to address complex population genetic challenges.

## 1 INTRODUCTION

High rates of species extinction and large declines in biodiversity are critical issues facing conservationists today (Fa, Funk, & O'Connell, 2011). Severe and prolonged population bottlenecks are perhaps the most damaging factors in these declines, leading to reduction in allelic diversity and increased inbreeding (Allendorf, Luikart, & Aitken, 2012; Markert et al., 2010). A powerful method for mitigating genetic diversity loss is genetic rescue. Genetic rescue occurs when population demographic rates are improved following the introduction of new genetic material into small and/or inbred populations (Whiteley, Fitzpatrick, Funk, & Tallmon, 2015). A recent review of genetic rescue data indicated that 92.9% of recipient populations showed a fitness benefit, such as increased fecundity and reduced inbreeding coefficients (Frankham, 2015). Genetic rescue is still rarely applied in conservation due to concerns over outbreeding depression, potential demographic disruption (Frankham, 2015) and a greater focus on threat abatement (Pierson et al., 2016), but there have been several iconic success stories such as the wolf *Canis lupus *(Vilà et al., 2003) and Florida panther *Puma concolor* (Hostetler, Onorato, Jansen, & Oli, 2013).

The use of genetic rescue remains challenging if ideal source populations do not exist. An ideal source population should inhabit a similar environment to the recipient population, have exchanged genes within the last 500 years and have no fixed chromosomal differences (Frankham et al., 2011). The present literature discusses the trade‐off between a potential source that is genetically similar but in different habitat from the recipient, versus a potential source that is genetically differentiated but in a more ecologically similar environment (e.g., Fitzpatrick et al., 2016; Kronenberger et al., 2017). Choosing to conduct “non‐ideal” genetic rescue will benefit from examining multiple data and analysis types. For example, a combination of microsatellite data and pedigree reconstruction revealed that a reciprocal translocation between two inbred populations of South Island robin *Petroica australis* provided genetic rescue in both (Heber et al., 2013). Furthermore, an increase in genetic diversity among hybrid offspring, relative to inbred offspring, was seen at low‐diversity immune genes (Toll‐like receptors) but not at high‐diversity immune regions (major histocompatibility complex) (Grueber, Sutton, et al., 2017). Another useful resource to inform genetic rescue planning is stochastic population modelling (e.g., Vortex [Lacy & Pollak, 2014], AlleleRetain [Weiser, Grueber, & Jamieson, 2012]). Stochastic population models can predict rates of genetic diversity loss based on population parameters and compare the effectiveness of alternative management strategies (such as supplementation rates) for maintaining diversity (Weiser, Grueber, & Jamieson, 2013). Decision‐making that utilizes both molecular genetic data and simulation modelling can inform action to prevent further genetic diversity loss in at‐risk populations (Buckland et al., 2014; Pelletier et al., 2017).

One species suffering from a persistent threat is the Tasmanian devil, *Sarcophilus harrisii*. Over the last 20 years, the devil population has declined by 77% due to a transmissible cancer, devil facial tumour disease (DFTD) (Hawkins, Baars, Hesterman, Hocking, & Jones, 2006; Lazenby et al., 2018; McCallum, Tompkins, Jones, Lachish, & Marvanek, 2007). Devils are a crucial component of the Tasmanian ecosystem, so their loss would likely result in complex ecological shifts (Fancourt, Hawkins, Cameron, Jones, & Nicol, 2015; Hollings, Jones, Mooney, & McCallum, 2016). Low genetic diversity has characterized the devil population for at least 100 years (Miller et al., 2011; Morris, Austin, & Belov, 2013), further exacerbated by recent population fragmentation due to DFTD (Hendricks et al., 2017). Ongoing devil management needs to occur in a landscape from which the prevailing threat, DFTD, cannot be fully mitigated at present, although research into managing the disease is ongoing (e.g., Kreiss, Brown, Tovar, Lyons, & Woods, 2015). DFTD remains a salient challenge to devil conservation, with the discovery of a second transmissible cancer in the species (Pye et al., 2015), and recent evidence that certain devil genotypes may provide a survival advantage in the presence of DFTD (Epstein et al., 2016; Pye et al., 2016; Wright et al., 2017). It is possible that evolutionary rescue in the face of DFTD (i.e., population recovery via adaptation to the prevailing threat; Carlson, Cunningham, & Westley, 2014) may occur in devil populations, although the diagnostic “third phase” (i.e., demographic recovery) is yet to be confirmed across the landscape (Lazenby et al., 2018). Meanwhile, a greater understanding of the necessity for genetic rescue of wild populations is timely.

This study was developed to assess the need for genetic rescue in three wild devil populations at the request of the species’ conservation managers. Few objectively “ideal” options for genetic rescue of wild devil populations remain. There is little evidence of large, outbred populations from which new diversity can be sourced, although we note that the full range of devils is not regularly surveyed, and opportunistic sampling suggests that unsurveyed populations may hold greater diversity (e.g., O'Connor, 2016). In the meantime, devils must be sourced from the large conservation breeding programme (the “insurance population”; Hogg et al., 2015) or isolated wild reserves such as offshore islands (including Maria Island; Thalmann et al., 2016). These potential sources were themselves derived from limited wild sites, so their diversity is also low. The questions posed by the conservation management team were around which populations to prioritize for genetic rescue, as opposed to where the devils should be sourced from. Here, we generate genetic rescue recommendations for three wild devil populations, in the face of a prevailing threat, by combining four major analysis types:

1. Putatively neutral genetic diversity by genotyping 31 microsatellites (following Gooley, Hogg, Belov, & Grueber, 2017; Jones, Paetkau, Geffen, & Moritz, 2003). For one site, neutral diversity data were collected at two time‐points, 10 years apart (before and after DFTD arrived at the site; see Methods).
2. Genetic diversity linked to functional regions by genotyping 11 MHC‐linked microsatellite loci (following Cheng & Belov, 2012). In many vertebrates, MHC diversity plays an important role in immune response (Radwan, Biedrzycka, & Babik, 2010) and mate choice (Kamiya, O'Dwyer, Westerdahl, Senior, & Nakagawa, 2014).
3. Quantitative data on the demographic effects of DFTD in wild devil populations, based on previous analysis of 20 years of field data from DFTD‐present and DFTD‐absent populations across Tasmania (Grueber, Fox, Belov, Pemberton, & Hogg, 2018; Lazenby et al., 2018).
4. Genetic diversity projections, by conducting stochastic population simulations of rare diversity in populations of various sizes and determining the impact of genetic supplementation on allelic diversity (following Weiser et al., 2013).

We predict that genetic diversity of our three study populations will be low and connectivity poor. Due to the small population sizes, we predict that stochastic modelling will project further declines in diversity, so we use our models to explore levels of supplementation that could mitigate this loss. We integrate field observations of the demographic effects of DFTD into our population modelling, to test whether processes that may influence effective population size (such as changes in breeding rates due to DFTD) impact diversity retention. More generally, our analysis serves as a model for combining multiple data and analysis types to assess genetic rescue options in an applied conservation context.

## 2 MATERIALS AND METHODS

### 2.1 Focal study populations

**Figure 1:**
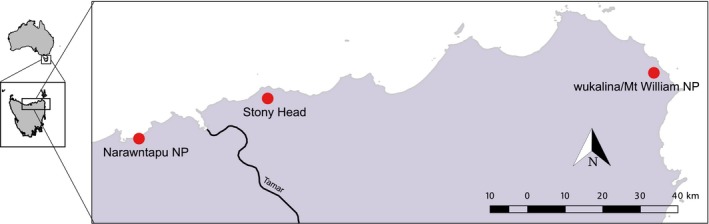
Devil populations in consideration for genetic rescue (points) in Tasmania, and the location of the Tamar River, a potential barrier to movement

In mid‐2014, staff from the Save the Tasmanian Devil Program (STDP) collected devil samples from wild populations at Narawntapu NP (National Park; *N* = 17), Stony Head (*N* = 24) and wukalina/Mt. William NP (*N* = 19) (Figure 1). Trapping was undertaken by the STDP under their standard operating procedures as part of ongoing monitoring of the species, and ear‐punch biopsy samples (collected into 70% ethanol) shared with the University of Sydney for genetic analysis. Full sampling protocols are reported elsewhere (e.g., Hawkins et al., 2006). Although our sample sizes are small, the populations from which each sample is obtained are similarly small. At the time of sampling, capture–mark–recapture estimated population size at Narawntapu NP was 19 animals (95% CI: 14, 31), at Stony Head 11 animals (95% CI: 7, 22) and at wukalina/Mt. William NP 12 animals (95% CI: 9, 16) (Lazenby et al., 2018). Therefore, it is likely that the animals included here are representative of the populations they are sampled from. Further details about our study populations can be found in Supporting Information (“Methods S1”). In addition to the recent samples, there was an opportunity to test for a change in diversity at the Narawntapu NP site, as previous microsatellite data from that population were available (*N* = 11 devils, 10 loci [Hogg et al., 2015; Jones, Paetkau, Geffen, & Moritz, 2004]). These previous data are referred to herein as “Narawntapu NP 2004,” and were collected prior to DFTD arrival at the site, which occurred in approximately 2006 (Lazenby et al., 2018).

### 2.2 Molecular genotyping and analysis

DNA was extracted using a standard phenol/chloroform method with ethanol precipitation (Sambrook, Fritsch, & Maniatis, 1989) and stored at –20°C. Samples were genotyped at 42 microsatellite loci previously developed for the Tasmanian devil: 31 putatively neutral loci (Gooley et al., 2017; Jones et al., 2003), plus nine loci known to be linked to MHC Class I and two loci linked to MHC Class II (Cheng & Belov, 2012). For Narawntapu NP, we compare newly collected microsatellite genotypes with the earlier data set. Not all samples from 2004 were available, so a small subset (*N* = 4) were re‐amplified as positive controls to standardize scoring. Microsatellite genotyping protocols are provided in Supporting Information (“Methods S1”).

Heterozygosity statistics (*H_O_* and *H_E_*, observed and expected heterozygosity, respectively) and tests for deviation from Hardy–Weinberg equilibrium were evaluated using Arlequin v3.5 (Excoffier & Lischer, 2010). *N_A_*, number of alleles, and *A_R_*, allelic richness controlling for variation in sample sizes, were evaluated using the basicStats function of the diveRsity package (Keenan, McGinnity, Cross, Crozier, & Prodöhl, 2013) for R v3.3.1 (R Core Team, 2017). Pairwise population diversity comparisons using *G'*_ST_ (Hedrick, 2005) were evaluated using the fastDivPart function of the diveRsity package, and its confidence interval was estimated by 1,000 bootstrap iterations.

The 2004 Narawntapu NP population was genotyped prior to development of additional MHC‐linked and neutral microsatellites, so comparison between the two sampling time‐points for this site was based on only 10 neutral loci. The two time‐points were compared in terms of *A_R_*, *H_O_* and *H_E_* at each locus, by fitting generalized linear mixed models using the R package lme4 v1.1–12 (Bates & Maechler, 2009). The diversity measures were the response variables, year (2004 [reference category] or 2014) was a binary fixed factor, and locus ID was fitted using random intercepts. Full modelling details are provided in Supporting Information (“Methods S1”).

Structural relationships among the sampled individuals were also inferred using discriminant analysis of principal components (DAPC; Jombart, Devillard, & Balloux, 2010) with the R package adegenet (Jombart, 2008). For all analyses, parameter estimates were considered statistically significant at *α* = 0.05 if their 95% confidence intervals (obtained from resampling methods or as 1.96 × standard errors from modelling results) excluded zero.

### 2.3 Stochastic simulation of allelic diversity retention

We used the R package AlleleRetain v2.0.2 (Weiser et al., 2012) to project the probability of each population retaining rare allelic diversity with and without varying levels of supplementation. AlleleRetain simulates neutral allelic diversity and therefore models diversity loss via genetic drift. Maintaining allelic diversity requires larger population sizes and/or greater connectivity than would be required to avoid other small population genetic issues, such as accumulation of inbreeding (Allendorf, 1986; Allendorf et al., 2012; Frankham et al., 2017). Thus, although inbreeding is likely to be rapidly accumulating in our simulated populations, we focus on loss of allelic diversity.

Full details of parameters used for AlleleRetain are provided in Supporting Information Table S1, and a summary of models explored is provided in Supporting Information Table S2. In general, models were parameterized based on field observations at each site (DPIPWE unpubl. data; Lazenby et al., 2018). We also utilized the findings from wild devil populations across Tasmania to account for demographic differences between sites with and without DFTD (Grueber et al., 2018; Lazenby et al., 2018). Differences include different age structures (devils were younger in DFTD‐present sites), greater probability of females breeding and larger litter sizes in DFTD‐present sites, relative to DFTD‐absent sites (Grueber et al., 2018; Lazenby et al., 2018). DFTD‐present sites were also modelled with decreased survival rates relative to DFTD‐absent sites.

Simulations were run for 50 years, approximately 20 devil generations, in accordance with devil management planning (Metapopulation Advisory Committee, 2016). Each iteration modelled one locus, and alleles modelled in all populations were assumed to have the same starting diversity; that is, they were drawn from a population with two alleles, with a “rare” allele frequency taken as 5%. Rare alleles at or below 5% frequency were frequently seen for both the neutral and MHC loci we genotyped (see Results). We consider currently neutral alleles, as these alleles may be easily lost by genetic drift in small populations, but may also provide adaptive potential in future (Slade & McCallum, 1992). Our genetic management goal was a > 95% probability of retaining 90% of rare alleles (following Dussex & Robertson, 2018; Liu et al., 2014; Tracy, Wallis, Efford, & Jamieson, 2011; Weiser et al., 2013). This goal was said to be met if the 95% confidence interval for the probability of retaining a rare allele, over 1,000 replicates, was completely above 0.9 after 50 years. It is possible that diversity may be further lost after this time, but we used a 50‐year threshold as this is similar to current devil metapopulation management strategy (Metapopulation Advisory Committee, 2016). We modelled two sets of populations:

1. *Real populations*. The real populations were the same as our molecular studies: Narawntapu NP (starting size = 19; population ceiling = 75), Stony Head (starting size = 11; population ceiling = 100) and wukalina/Mt. William NP (starting size = 12; population ceiling = 150). Population ceilings were based on the size of the area and devil density in disease‐free sites (Guiler, 1970; Pemberton, 1990). These are likely to be overestimates, as they are not strict population sizes as described by Lazenby et al. (2018). We assumed population ceiling was constant. These populations currently have DFTD, so were modelled with “DFTD‐present” parameters (Table S1).
2. *Hypothetical populations*. We simulated hypothetical populations of starting size = 10, 20, 50, 100 or 200 devils, and population ceiling = 50, 100, 200, 500 or 1,000 devils. Hypothetical populations were simulated both assuming DFTD presence and DFTD absence, where the latter might represent managed populations free of disease (such as on islands or fenced areas).

All populations were assumed to be isolated from all other devil populations, apart from any management‐assisted supplementation (see below). Results are averaged over those replicates in which the simulated population did not go extinct (allelic diversity after 50 years can only be calculated for extant populations).

### 2.4 Simulation of management options

The effects of two types of management options, on neutral genetic diversity (alleles of frequency *q*_0_ = 0.05), were modelled: one‐off supplementation of devils in the second year of the simulation or ongoing (two‐yearly) supplementations of devils. We modelled one‐off supplementations of sizes 0, 14 (an operationally feasible number), 28 or 56 animals, and ongoing supplementations of size 0, 2, 4, 6, 8, 10, 12 or 14 animals every 2 years. For smaller population sizes, the higher levels of supplementation would lead to very high proportions of immigrants. We assumed an equal sex ratio of translocated animals and 90% survival following translocation. We also assumed that animals were sourced from a population that was not genetically differentiated from the recipient population. We assumed that, after any initial mortality associated with translocation, the introduced animals would show equal probability of breeding and survival as locally produced animals of the same age and sex. In a follow‐up set of models, migrants were given priority over locals in the breeding pool (using the AlleleRetain option mpriority = TRUE).

## 3 RESULTS

### 3.1 Population molecular diversity

Genetic diversity of each population was low (Table 1). After sequential Bonferroni correction, only two loci (in one population each) showed a statistically significant deviation from Hardy–Weinberg equilibrium (Table S3). As there were no consistent patterns of deviation, all loci were retained for analysis. Similar numbers of alleles and rates of heterozygosity were observed across neutral and MHC‐linked loci (Table 1). Approximately 10% of the allele frequencies for polymorphic loci within populations were ≤0.05 (9.4% for MHC and 10.3% for neutral alleles; Table S4).

**Table 1:** Microsatellite diversity statistics for Tasmanian devils sampled from three sites

	Population^a^	*N*	*N*_loci_ b	*N*_A_ (*SD*)	*A*_R_ (*SD*)	*H*_O_ (*SD*)	*H*_E_ (*SD*)
All loci	Narawntapu NP 2004	11	10	3.10 (0.99)	3.10 (0.99)	0.536 (0.236)	0.526 (0.135)
	Narawntapu NP 2014	17	42 (1)	2.79 (1.09)	2.77 (1.08)	0.504 (0.209)	0.472 (0.154)
	Stony Head	24	42 (3)	2.74 (1.06)	2.67 (1.00)	0.461 (0.195)	0.479 (0.177)
	wukalina/Mt William NP	19	42 (3)	2.93 (1.31)	2.84 (1.27)	0.449 (0.185)	0.483 (0.187)
MHC only	Narawntapu NP 2004	0					
	Narawntapu NP 2014	17	11	3.09 (1.58)	3.09 (1.58)	0.582 (0.196)	0.515 (0.146)
	Stony Head	24	11	3.00 (1.10)	2.97 (1.07)	0.546 (0.192)	0.531 (0.154)
	wukalina/Mt William NP	19	11	3.55 (1.51)	3.48 (1.46)	0.494 (0.162)	0.575 (0.085)
Neutral only	Narawntapu NP 2004	11	10	3.10 (0.99)	3.10 (0.99)	0.536 (0.236)	0.526 (0.135)
	Narawntapu NP 2014	17	31 (1)	2.68 (0.87)	2.66 (0.85)	0.475 (0.210)	0.456 (0.156)
	Stony Head	24	31 (3)	2.65 (1.05)	2.56 (0.97)	0.427 (0.188)	0.458 (0.183)
	wukalina/Mt William NP	19	31 (3)	2.71 (1.19)	2.61 (1.14)	0.432 (0.193)	0.447 (0.205)

Notes

*N*: number of animals sampled; *N*_A_: number of alleles; *SD*: standard deviation across loci; *A*_R_, allelic richness; *H*_O_: mean observed heterozygosity; *H*_E_: mean expected heterozygosity.

a Estimated population sizes Narawntapu NP = 19 (95% CI: 14, 31), Stony Head = 11 (95% CI: 7, 22), wukalina/Mt. William NP = 12 (95% CI: 9, 16)

b Where shown, number in parenthesis indicates number of monomorphic loci excluded from heterozygosity statistics.

**Figure 2:**
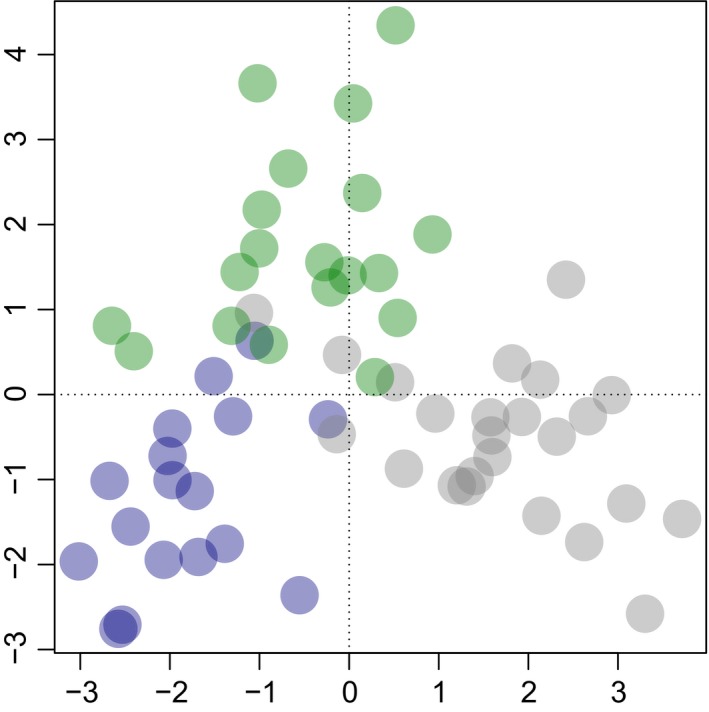
First two axes of DAPC of three populations of Tasmanian devils (blue = Narawntapu NP, grey = Stony Head and green = wukalina/Mt William NP) based on 42 microsatellite loci. Similar plots based on only neutral or MHC‐linked microsatellite loci are provided at Figure S1

Considering the recent sampling, all three populations showed moderate differentiation from one another (via both *G'*_ST_ [Table 2] and DAPC [Figure 2] analysis), suggesting low connectivity. When considering neutral or MHC‐linked loci separately, population differentiation estimated under *G'*_ST_ was similar to the overall values for neutral loci, but slightly higher for MHC‐linked loci (Table 2), although this pattern was not clearly reflected by DAPC analysis (Figure S1). The Narawntapu NP declined in diversity from 2004 to 2014, a pattern that was statistically significant at *α* = 0.05 for allelic richness (Table 3; confidence intervals for the slope estimate for *A_R_* excludes zero), but not for the two heterozygosity statistics (Table 3). The 2004 and 2014 samples showed moderate, statistically significant differentiation (*G'*_ST_ = 0.098, Table 2).

**Table 2:** Bias‐corrected estimates of *G'*_ST_ (Hedrick, 2005) with 95% confidence intervals evaluated using 1,000 bootstraps in diveRsity (Keenan et al., 2013)

		Narawntapu NP 2014	Stony Head	wukalina/Mt William NP
All loci	Narawntapu NP 2004	0.098 (0.062, 0.148)	0.116 (0.084, 0.162)	0.122 (0.09, 0.163)
	Narawntapu NP 2014		0.141 (0.089, 0.201)	0.114 (0.063, 0.178)
	Stony Head			0.100 (0.067, 0.142)
MHC only	Narawntapu NP 2004	—	—	—
	Narawntapu NP 2014		0.191 (0.084, 0.317)	0.135 (0.045, 0.263)
	Stony Head			0.142 (0.074, 0.231)
Neutral only	Narawntapu NP 2004	0.106 (0.068, 0.167)	0.126 (0.089, 0.175)	0.131 (0.1, 0.176)
	Narawntapu NP 2014		0.125 (0.076, 0.185)	0.108 (0.065, 0.161)
	Stony Head			0.088 (0.056, 0.125)

**Table 3:** Generalized linear mixed modelling results evaluating the change in Tasmanian devil heterozygosity, at 10 putatively neutral microsatellite loci, from 2004 (reference category) to 2014 at the Narawntapu NP site. All models contained a random intercept for “locus.”

Response	Predictor	Estimate (*SE*)	95% CI
*A*_R_	Intercept	1.086 (0.088)	0.913, 1.258
	Year (2014)	−0.218 (0.079)	−0.373, −0.064
*H*_O_	Intercept	0.152 (0.262)	−0.361, 0.665
	Year (2014)	−0.383 (0.254)	−0.882, 0.115
*H*_E_	Intercept	0.109 (0.173)	−0.231, 0.448
	Year (2014)	−0.381 (0.198)	−0.769, 0.007

Note

*N*_A_: number of alleles (Poisson error structure); *H*_O_: observed heterozygosity (binomial error structure); *H*_E_: expected heterozygosity (logit transformed).

### 3.2 Projected retention of allelic diversity

**Figure 3:**
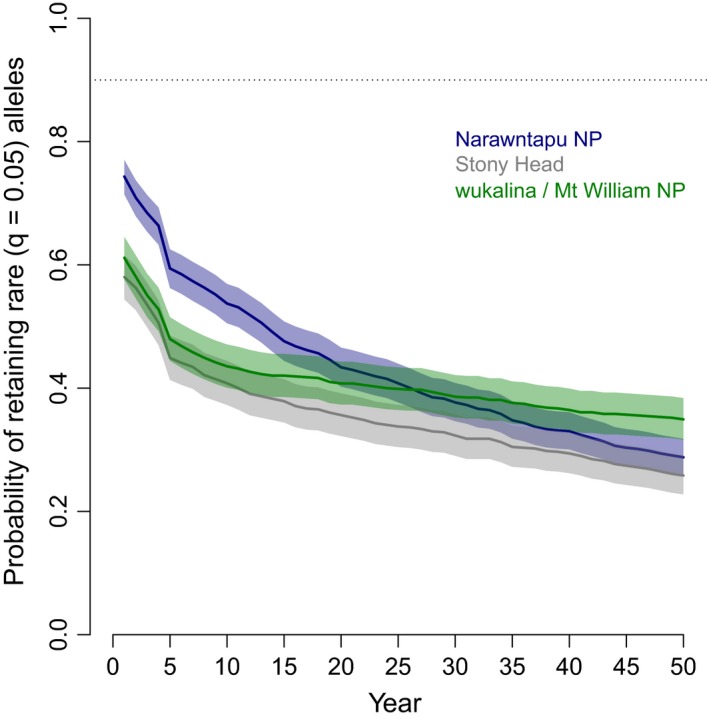
Proportion of rare alleles retained in three wild devil populations (as shown in legend) after 50 years, with no immigration. Solid lines indicate the mean population estimate over 1,000 iterations, with the shaded area the 95% confidence intervals. The dashed line indicates the 90% retention goal

Populations at the end of each simulation had age and reproductive structures that were consistent with our input parameters (Figure S2). Because we modelled isolated, finite populations, all lost diversity, and without immigration, none met the goal of retaining 90% of rare alleles after 50 years (Figure 3). The probabilities of simulated populations persisting to the end of the simulation without immigration were as follows: Narawntapu NP 0.966 (95% CI 0.952; 0.976), Stony Head 0.755 (0.727; 0.781) and wukalina/Mt. William NP 0.790 (0.763; 0.815).

**Figure 4:**
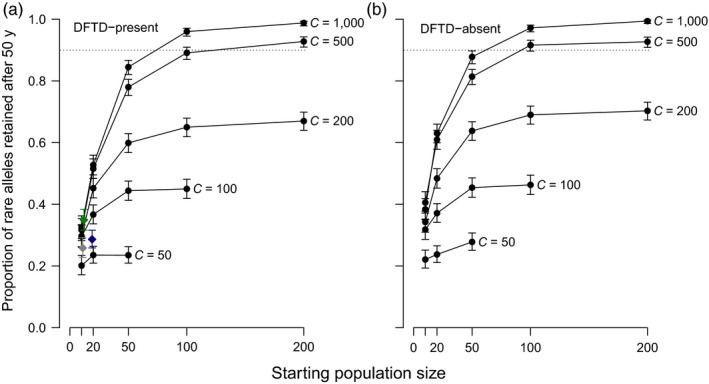
Effect of starting population size and population ceiling (“C”) on the proportion of rare alleles retained in devil populations after 50 years, whether DFTD is present (a) or absent (b). Each data point is the proportion of rare alleles retained at 50 years; error bars are the 95% confidence limit based on 1,000 replicates. Five population ceiling values are shown; the dashed line indicates the 90% retention goal. In (A), the coloured diamonds represent the proportion of rare alleles remaining in simulated populations of the three wild populations (blue = Narawntapu NP, grey = Stony Head and green = wukalina/Mt William NP) at the end of 50 years (i.e., the end‐points of Figure 2)

In the absence of management, only very large populations would be expected to meet the genetic goal (Figure 4). Whether simulated populations were parameterized assuming demographic rates for DFTD presence (Figure 4a) or DFTD absence (Figure 4b) had little effect on the expected retention of rare alleles, although simulated DFTD‐absent sites retained marginally more diversity (Figure 4). Without management, the genetic goal of retaining over 90% of rare neutral alleles for 50 years could only be met at a site with a population that grows to a ceiling of at least 500, and only if it is started with at least 200 individuals (Figure 4). A site with a population ceiling of 1,000 could retain diversity for 50 years if the population was started with at least 100 individuals (Figure 4).

**Figure 5:**
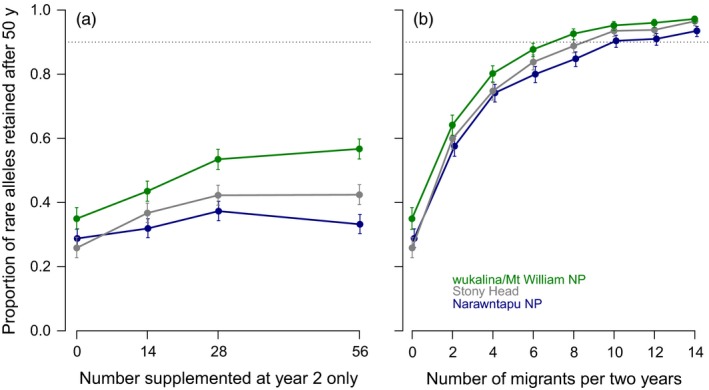
Effect of an initial, one‐off supplementation (a) or two‐yearly ongoing supplementation (b), at varying rates, on the retention of genetic diversity in three wild devil populations (as shown in legend). Each data point is the proportion of rare alleles retained after 50 years; error bars are the 95% confidence limit based on 1,000 replicates. The dashed line indicates the 90% retention goal. (Note that, for clarity, data points have been “offset” slightly with respect to the *x*‐axis)

For our three real populations, none of the one‐off supplementation options (up to 56 animals) were sufficient to maintain 90% of rare alleles after 50 years (Figure 5a). Where migrants were assumed to have the same likelihood of breeding as incumbent animals, ongoing supplementation was effective at maintaining neutral rare alleles in recipient populations: The population at wukalina/Mt. William NP would retain diversity with eight animals every two years, Stony Head with 10 animals every two years and Narawntapu NP with 14 animals every two years (Figure 5b). Models assuming that immigrants were given preference to breed indicated similar results for one‐off supplementation (Figure S3a), while slightly fewer animals were required to maintain neutral rare alleles via ongoing supplementation (wukalina/Mt. William NP required six animals every two years, Stony Head eight animals every two years and Narawntapu NP 10 animals every two years; Figure S3b). Note that, in all supplementation models, migrants were considered to come from a population with the same focal allele frequency (*q* = 0.05) as the starting condition of the recipient populations.

Variation in supplementation requirements for each population is largely attributable to differences in population ceiling, as starting population sizes varied little (wukalina/Mt. William NP 12 [ceiling* *= 150]; Stony Head 11 [ceiling = 100], Narawntapu NP 19 [ceiling = 75]) and one‐off supplementations at the beginning had little effect after 50 years (Figure 5a). Supplementation (either one‐off or ongoing) improved the probability that our simulated populations persisted to the end of the simulation (Figure S4).

## 4 DISCUSSION

This study was developed in response to a series of management questions from the Save the Tasmanian Devil Program given the persistence of wild devil populations with DFTD (Lazenby et al., 2018), but these types of questions are not unique to devils. Where should a management response start? Should populations be supplemented, and if so, which ones to target first, how often and with how many? Globally, conservation managers tasked with maintaining wild populations pose all these questions, and more. The breadth of data available for devils allows us to show how multiple data analysis types can be used to provide genetic recommendations for the supplementation of wild sites.

### 4.1 Devil populations have low diversity and poor connectivity

The microsatellite data presented support previous reports of low genetic diversity in Tasmanian devils (Cheng, Sanderson, Jones, & Belov, 2012; Jones et al., 2004; Morris, Wright, Grueber, Hogg, & Belov, 2015; Murchison et al., 2012; Wright et al., 2015) and reports of low‐moderate differentiation among sites (Hendricks et al., 2017). Although our sample sizes were small, recent studies have shown that small sample sizes in conservation genetics do not systematically bias point estimates of population differentiation and expected heterozygosity, although allelic diversity may be underestimated (Hale, Burg, & Steeves, 2012; Smith & Wang, 2014). We therefore expect that our population comparisons, particularly *G'*_ST_ and *H_E_*, likely reflect true patterns. Neutral and MHC patterns were similar: showing low diversity and poor connectivity. If our three study populations remain isolated, they risk losing further genetic diversity due to their small sizes. Temporally separated samples from Narawntapu NP indicate that some diversity may have already been lost, as allelic richness declined. Trends for other measures of diversity, namely heterozygosity, were also in a negative direction, although not statistically significant, possibly because few loci were available for comparison (*N = *10 loci). These molecular data support the argument that, from a conservation planning point of view, all three sites are in equivalent need of genetic rescue.

### 4.2 Stochastic models illustrate the necessity of genetic management

All small populations will lose diversity via prolonged population bottlenecking if they remain isolated (Allendorf et al., 2012). This analysis has identified thresholds for the retention of rare, neutral diversity for devil populations. None of our three “real” populations nor most of our realistic “hypothetical” populations are expected to provide a 95% probability of retaining at least 90% of rare alleles after 50 years, if they remain isolated (Figure 4). A population smaller than 500 – 1,000 animals (depending on the starting size) would require ongoing management if diversity loss is to be avoided. These results are largely consistent with theoretical expectations that an effective population size *N*_e _> 500 is required to maintain adaptive potential in the long term, in the absence of management (Frankham, Bradshaw, & Brook, 2014). The importance of final (i.e., maximal) population size in our analysis is illustrated by examining populations where the starting size equals the final size (Figure 4). Starting closer to the final size is insufficient to retain 90% of rare alleles after 50 years if the final population size is low (≤200; Figure 4). Ongoing supplementation is more effective at maintaining diversity in devils than one‐off supplementation (Figure 5), consistent with studies of other species (e.g., Weiser et al., 2013).

Although demographic differences are seen between DFTD‐present and DFTD‐absent populations (Grueber et al., 2018; Lachish, McCallum, & Jones, 2009; Lazenby et al., 2018), our simulations suggest that, for a given starting and final population size, these differences do not translate to large differences in the retention of neutral diversity (Figure 4). In the wild, an important difference between DFTD‐present and DFTD‐absent populations is their propensity for population growth. Newly established DFTD‐absent populations, such as the introduction of healthy devils to Maria Island (McLennan et al., 2018; Rogers, Fox, Pemberton, & Wise, 2016; Thalmann et al., 2016), grow very quickly. Conversely, the arrival of DFTD in a population leads to rapid population decline and low population densities (Lazenby et al., 2018). Thus, an appropriate point of comparison for DFTD‐present versus DFTD‐absent populations would be DFTD‐present populations with a size ≤ 50, versus DFTD‐absent populations of size ≥100 (Figure 4). In other words, the maintenance of neutral diversity depends more on population size than the presence or absence of DFTD. This conclusion is consistent with population genetic theory, which predicts that the loss of genetic diversity depends primarily on the severity and duration of a population bottleneck (Allendorf et al., 2012).

### 4.3 Considerations regarding the supplementation modelling of devil populations

Like all models that approximate natural populations, ours have limitations, and we have made simplifying assumptions to provide a starting point for management planning and ranking priorities (CBSG, 2008; Peck, 2004). Our models examining supplementation options for devils are focussed on genetic rescue, but also raise some interesting challenges due to the complexity of devil management in the DFTD‐affected landscape.

The stochastic modelling conducted here targets rare, neutral alleles. Our predictions based on allelic diversity are conservative with regard to the management of inbreeding, which can be minimized with lower rates of translocation (Allendorf et al., 2012). We have not considered the effects of supplementation on putatively functional diversity. Data from wild devil populations suggest that certain devil genotypes may provide a survival advantage in the presence of DFTD (Epstein et al., 2016; Pye et al., 2016; Wright et al., 2017). We do not currently know what the consequence of releasing devils into the DFTD‐affected landscape will be, and so any supplementations should be closely monitored to detect any effects on DFTD epidemiology.

We do not specifically examine the characteristics of source populations, assuming them to be not genetically differentiated from the modelled recipient populations. This is not an unreasonable assumption, as migrant devils are currently sourced from other managed sites across the insurance population, including subpopulations that were established from, and periodically supplemented by, devils from the wild (Hogg, Lee, Srb, & Hibbard, 2017). It is also worth noting that “supplementation” does not necessarily demand active translocations from managed sites. Activities that facilitate natural devil movements among sites may have the same outcome from the perspective of genetic rescue. The feasibility of improving natural connectivity between wild sites will depend on the characteristics of each population and the ease with which animals can migrate.

We have considered two scenarios of migrant behaviour: their breeding at rates similar to locals, versus priority breeding over locals. This simple comparison showed little difference in the impact of migrants on diversity. However, uncertainties do remain, which should be examined through follow‐up field monitoring. For example, natural migration of very small numbers of individuals can result in substantial genetic contributions to recipient populations (e.g., Åkesson et al., 2016; Gustafson, Vickers, Boyce, & Ernest, 2017). Similarly, if migrants contribute at a lower rate than locals (Waters, Fraser, & Hewitt, 2013), a greater number of animals may need to be translocated to maintain diversity. Together, follow‐up monitoring will be essential to understanding migrant behaviour following translocation into incumbent populations.

Despite the aforementioned caveats, an advantage of our analysis is that all models are informed by extensive field data, providing precise estimates of demographic parameters (Grueber et al., 2018; Lazenby et al., 2018). In less well‐studied systems, such extensive demographic data are not available. Where parameter estimates are uncertain, sensitivity analysis is advisable and can be used to help identify those parameters that have the greatest influence on the modelled outcomes (Naujokaitis‐Lewis, Curtis, Arcese, & Rosenfeld, 2009; Peck, 2004).

### 4.4 Genetic management of wild devil populations

Genetic rescue of threatened populations is challenging, presenting many considerations and risks. For example, it is difficult to predict whether increased connectivity will facilitate or impede evolutionary rescue (see Introduction), as the latter is contingent on demographic, genetic and extrinsic factors, and interactions thereof (Carlson et al., 2014). Nevertheless, our results for devils reveal wild populations in need of genetic rescue. Harnessing the power of multiple analysis types enables us to obtain multiple perspectives on this problem, each of which point in the same direction. Functional and neutral diversity statistics show that diversity is low and connectivity poor; temporal sampling shows decreasing diversity over a short time frame; stochastic modelling suggests that these issues are likely to continue to worsen; and the integration of field observations of DFTD with our stochastic models shows that population size is the primary determinant of genetic diversity loss. Together, these observations provide an argument for improving devil population connectivity and/or immigration for the purposes of retaining genetic diversity.

In the spirit of the “Tools & Tech” project (Hogg et al, 2017), the findings herein were provided to the Save the Tasmanian Devil Program in 2015 to inform their decisions regarding genetic supplementation of the three sites in this study. In September 2015, 20 captive devils were released to Narawntapu NP (Grueber, Reid‐Wainscoat, et al., 2017); in August 2016, 16 captive and 17 Maria Island devils were released to Stony Head (Fox & Seddon, in press); and in May 2017, 33 Maria Island devils were released to wukalina/Mt. William NP (Fox & Seddon, in press). In addition to population supplementation, devils were released for a variety of reasons, such as determining the survival of released animals (e.g., Grueber, Reid‐Wainscoat, et al., 2017) and testing the efficacy of immunization against DFTD (Pye et al., 2018; Fox & Seddon, in press). The Stony Head release currently provides the best data on the outcomes of supplementation. DPIPWE trapping trips conducted in the three years prior to the release, and one year after the release to Stony Head, showed that sex ratio and female productivity were similar before and after the release (Supporting Information Table S5). However, longer term monitoring is required to determine whether genetic rescue has been realized, and parentage analysis of offspring will show the degree to which the immigrants contribute to the population (e.g., Åkesson et al., 2016; Hasselgren et al., 2018; Heber et al., 2013; Gustafson et al., 2017). Ongoing molecular analysis will thus be essential to determine whether supplementation intended to mitigate one threat (namely the loss of genetic diversity) also retains resilience despite uncertainty surrounding other threats (namely DFTD), or whether further supplementation should be avoided. Nevertheless, the results of the current study demonstrate that the avoidance of supplementation carries its own risks, and it is important to avoid letting uncertainty alone drive conservationists into a de facto decision to “do nothing” (Grueber, 2017; Meek et al., 2015; Mills, 2017; Tallmon, 2017). We strongly recommend that the decision‐making process outlined in the current study be tested by closely monitoring and critiquing the outcomes of past releases over five years (i.e., approximately two devil generations). The results will reveal whether it is appropriate to activate the ongoing supplementation recommendations presented herein.

Taken together, the current results reveal a system in need of genetic rescue. Analysing the consequences of supplementation activities will continue to inform conservation strategy in the face of complex prevailing threats.

## 5 DATA ARCHIVING STATEMENT

Data for this study are available at the Dryad Digital Repository: https://doi.org/10.5061/dryad.11ss226>

## CONFLICT OF INTEREST

None to declare.

Informed consent: Not applicable; non‐human research.
