# Supplementary material for: Complex problems need detailed solutions: Harnessing multiple data types to inform genetic management in the wild
Source: Evol Appl. 2018 Dec 26;12(2):280–91. doi: 10.1111/eva.12715 (PMC6346650; doi:10.1111/eva.12715)
Supplement: Supplementary file 1 [file EVA-12-280-s001.docx]

## Supporting information for: Grueber CE, Fox S, McLennan EA, Gooley RM, Pemberton D, Hogg CJ, Belov K “Complex problems need detailed solutions: Harnessing multiple data types to inform genetic management in the wild” *Evolutionary Applications*

## Supplementary methods

#### Focal study populations

All three of our study populations are at long-term diseased sites. DFTD itself was thought to have first emerged at wukalina / Mt. William NP in 1996 (Hawkins et al., 2006). Long-term diseased sites are typified by a population crash, and an eventual plateau at around 20 individuals in which a few females continue to breed, preventing population extinction (Lazenby et al., 2018).

Current genetic evidence suggests that Tasmanian devil populations show mild local, regional and state-wide structuring (Jones et al., 2004; Lachish et al., 2011; Miller et al., 2011), which has been exacerbated by the arrival of DFTD (Lachish et al., 2011). However, there is currently no evidence that any sub-populations are completely isolated (Huxtable et al., 2015). For the current study populations, the Tamar River presents a physical barrier to movement for devils from Narawntapu NP to the other two sites (Fig. 1).

#### Microsatellite genotyping

Microsatellites were amplified in 10 μl reactions containing 1 μl purified DNA (standardised to 10 – 20 ng/μl), 5 μl 2× Type-it Master Mix (Qiagen) and primer mix containing forward and reverse primers (ABI) to a final concentration of 0.2 μM. Primers for neutral loci were fluorescently tagged and multiplexed according to Gooley et al. (2017); primers for MHC loci were fluorescently tagged and amplified individually following Cheng and Belov (2012). Thermocycling conditions followed Qiagen Type-it guidelines, on a Bio-Rad T100 Thermal Cycler. Fragment sizes were resolved on an ABI 3130 Genetic Analyser with GeneScan 500 (LIZ) size standard; genotyping chromatograms were analysed and scored using Genemarker v1.95 (Soft Genetics LLC).

#### Temporal change in diversity at Narawntapu NP

To model the change in genetic diversity at Narawntapu NP we used linear regressions of the structure diversity ~ as.factor(year) + (1|locusID), where three measures of diversity were each separately modelled as the response variable. *A_R_* is a count-based metric, but non-integer, and therefore log transformed and fitted with a Gaussian model. *H_O_* was fitted as a binomial response (logit link) (number of genotyped individuals was the binomial denominator [trial] and observed number of heterozygotes the binomial numerator [event]. *H_E_* is a proportion-based metric, but not count-based, so this variable was logit transformed and the model fitted with a Gaussian model. Monomorphic loci were excluded from the two heterozygosity models.

**Table S1** Life history, demographic and population parameters used in AlleleRetain modelling for devils. Parameter values that differ between DFTD-present and DFTD-absent sites are indicated. For more detailed parameter descriptions, see the AlleleRetain user manual, available at <https://sites.google.com/site/alleleretain/>.

| Parameter | Brief description | Value(s) |
| --- | --- | --- |
| Source population | |  |
| q0 | Frequency of rare allele in the source population. | 0.05 |
|  |  |  |
| sourceN | Size of source population. | Infinite |
| Starting population | |  |
| startN | Number of starters; note that not all will become genetic founders. Values in parentheses are the lower and upper 95% CIs of the capture-mark-recapture, respectively. | 19 Narawntapu NP  11 Stony Head  12 wukalina / Mt. William NP |
| startAge | Age class ("juvenile", "young adult", or "adult") of starters, supplementals, and migrants. | Young adult |
| startSR | Sex ratio (proportion male) of starters, supplementals, and migrants. | 0.5 |
| exactSR | Whether startSR gives the exact sex ratio of individuals released (“true”) or sexes are assigned randomly based on the probability given by startSR (“false”). | False |
| Translocated individuals | |  |
| inisurv | Initial survival rate, as a proportion (range 0-1), of starters, supplementals, and migrants immediately post-release (separate from annual mortality). | 0.9 |
| addN | Numbers of individual (supplementals) to release, as a one-off, in years soon after population establishment | 0, 14, 28, 56 |
| addyrs | Year in which to release supplementals | 2 |
| migrN | Number of migrants to add at each interval given by migrfreq. | 0, 2, 4, 6, 8, 10, 12, 14 |
| migrfreq | Interval at which to add migrN migrants. | 2 |
| mpriority | Migrants are given priority over locally produced offspring to recruit into any available breeding vacancies. | False, True |
| removeL | Remove the corresponding number of locally produced adults to make room for migrants in the population when population is at K. | False |
| Characteristics of the established population | |  |
| K | Carrying capacity (population ceiling) | 75 Narawntapu NP 100 Stony Head 150 wukalina / Mt. William NP |
| Klag | Number of years for which population is held at or below initial size (breeding still occurs); indicates a prolonged bottleneck. | 0 |
| KAdults | Which animals are included in carrying capacity? Use “true” for K = number of adults or “false” for K = total individuals, including subadults, nonbreeders, etc. | False |
| reprolag | Number of years after establishment in which no reproduction occurs. | 0 |
| Life history traits of the simulated species | |  |
| mature | Average age (in years) at sexual maturity (first breeding); defaults to 1. | 2^1^ |
| matingSys | Mating system: "monogamy", "polygyny", or "polygynandry". In any case, pairs are formed and breed. With polygyny, each male can be part of more than one pair. | polygyny |
| matingLength | Determines whether individuals retain the same mate from year to year (“lifelong”) or not (“seasonal”). | Seasonal |
| meanMLRS | Mean lifetime reproductive success (LRS), in terms of number of matings that produce young (NOT number of offspring) a male gets over his lifetime. This is a population average for all males, including those that never reproduce, and may be a fraction. Each male is assigned an individual average from a gamma distribution with this mean and SD given by sdMLRS. The SD:mean ratio is more important than the magnitude of the mean. The individual mean indicates the male's “quality” and will be used to assess his chance of mating, relative to other males present, each year (i.e. does not translate directly into actual LRS experienced by that male). | 2.8 |
| sdMLRS | Among-male standard deviation in LRS. Used with meanMLRS as described above. | 0.8 |
| reproAgeM | List of ages at which males can breed. | 2, 3, 4, 5 |
| AgeOnMLRS | Expression describing the proportion of LRS achieved by a male at a particular age (for ages contained within reproAgeM). If a given age is not included in reproAgeM, reproductive output at that age will be set to 0 regardless of the value calculated by this equation. | -12.24 + 11.1*age - 2.7*age^2 + 0.2 *age^3 |
| nMatings | Average number of matings per female each year. | 1 |
| retainBreeders | Should established breeders retain their breeding status from year to year, and prevent young individuals from recruiting if the population is at K? Only used when matingSys = “monogamy”. | NA |
| MaxAge | Maximum allowable lifespan (in years); can be Inf. | DFTD-absent = 7  DFTD-present = 6 |
| SenesAge | Age (in years) after which annual survival will be reduced by senescence. Through this age, adult survival values are set according to adsurvivalF and adsurvivalM; after this age, annual survival decreases linearly to 0 at MaxAge. | DFTD-absent = 5  DFTD-present = 4^2^ |
| Expected demography of the new population | |  |
| adsurvivalF | Annual survival rate of adult females. | DFTD-absent = 0.85  DFTD-present = 0.80^3^ |
| adsurvivalM | Annual survival rate of adult males. | DFTD-absent = 0.85  DFTD-present = 0.80^3^ |
| nonbrsurv | Annual survival rate of nonbreeders (subadults [age 1-2] or adults that have not recruited). | DFTD-absent = 0.80  DFTD-present = 0.75^3^ |
| nonbrsurvK | Annual survival rate of nonbreeders when population is at K. | DFTD-absent = 0.70  DFTD-present = 0.65^3^ |
| juvsurv | First-year survival (from the stage described by youngperF, below, to the beginning of the next breeding season) when population is below K. | 0.5 |
| juvsurvK | First year survival when population is at K (used instead of juvsurv). | 0.5 |
| youngperF | Average number of offspring produced per mating each year per female. youngperF can be calculated for any reproductive stage (eggs, chicks, independent juveniles) as long as juvsurv indicates the proportion of individuals that survive from this stage to the beginning of the following breeding season. | DFTD-absent = 3.18  DFTD-present = 3.33^4^ |
| SDypF | Among-individual standard deviation of youngperF, e.g. 0.50 or 2. | 0.3 |
| ypF1 | Where younger breeders have reduced reproductive rates, this can be used to define the reproductive success for the first reproductive stage. | NA |
| ypF1yr | Max age at which ypF1 applies. | NA |
| MAXypF | Maximum annual number of offspring per individual. | 4 |
| MAXypFK | Maximum annual number of offspring per individual when population is at K. | 4 |
| ypFsex | Which member of a pair limits the female’s reproductive output for the year? | female |
| youngSR | Proportion of offspring that are male. | 0.5 |
| Simulation and output specifications | |  |
| trackall | Whether to track all individuals from the population through the simulation | False |
| GeneCount | Which individuals should be included (“all”, or only breeding “adults”) when the number of rare alleles is counted in the population each year. | All |
| nyears | Number of years to run the simulation. | 50 |
| nrepl | Number of replicates to run. | 1,000 |

^1^ Although DFTD-present sites have a higher rate of females breeding at 1 year (Lachish et al., 2009; Grueber et al., 2018), a lack of genetic parentage studies prevent us from making similar assumptions about males. As male and female maturity cannot be assigned separately by AlleleRetain, we have taken 2 years as an average in both DFTD-absent and DFTD-present sites.

^2^ Reduced by 1 year commensurate with the 1 year decrease in lifespan

^3^ Reduced by 5% (absolute) to account for lower ages at capture in DFTD-present sites (Grueber et al., 2018)

^4^ Based on productivity reported in Grueber et al., 2018; Lazenby et al., 2018

### Table S2 Overview of population simulations run in this study. The first three sets of models (named populations) were used to compare the effects of different types of supplementation on real populations. The models for hypothetical populations were used to compare genetic diversity retention in unmanaged populations of various sizes. All models were run for 50 years (approximately 20 devil generations) and considered the frequency of an allele with starting frequency of 0.05. See Table S1 for full model specifications.

|  | *N* | |  | Supplementation^1^ | |
| --- | --- | --- | --- | --- | --- |
| Population | Start | Max | DFTD? | One-off^2^ | Ongoing^3^ |
| Narawntapu NP | 19 | 75 | Yes | 0, 14, 28, 56 | 0, 2, 4, 6, 8, 10, 12, 14 |
| Stony Head | 11 | 100 | Yes | 0, 14, 28, 56 | 0, 2, 4, 6, 8, 10, 12, 14 |
| Wukalina / Mt. William NP | 12 | 150 | Yes | 0, 14, 28, 56 | 0, 2, 4, 6, 8, 10, 12, 14 |
| Hypothetical | 10, 20, 50, 100, 200 | 50 | Yes, No^4^ | 0 | 0 |
| Hypothetical | 10, 20, 50, 100, 200 | 100 | Yes, No^4^ | 0 | 0 |
| Hypothetical | 10, 20, 50, 100, 200 | 200 | Yes, No^4^ | 0 | 0 |
| Hypothetical | 10, 20, 50, 100, 200 | 500 | Yes, No^4^ | 0 | 0 |
| Hypothetical | 10, 20, 50, 100, 200 | 1,000 | Yes, No^4^ | 0 | 0 |

^1^ Only one type of supplementation was modelled at a time (i.e. when one type was set at >0, the other type was set to 0). Supplementation models were run twice: with and without migrants having priority breeding (see Methods).

^2^ One-off supplementation occurred in year 2 only.

^3^ Ongoing supplementation occurred at 2-yearly intervals from year 2

^4^ Models were run twice, with DFTD-present and DFTD-absent parameters (see Table S1 for details of how these differ)

### Table S3 Locus-level diversity data and tests of deviation from Hardy-Weinberg equilibrium for each population

| Pop Name | Locus | *N* | *N_A_* | *H_O_* | *H_E_* | p-value^1^ |
| --- | --- | --- | --- | --- | --- | --- |
| Narawntapu NP 2004 | Sh2g | 11 | 4 | 0.818 | 0.680 | 0.422 |
|  | Sh2i | 11 | 3 | 0.818 | 0.584 | 0.307 |
|  | Sh2L | 11 | 2 | 0.455 | 0.368 | 1.000 |
|  | Sh2p | 11 | 4 | 0.818 | 0.645 | 0.179 |
|  | Sh2v | 11 | 5 | 0.727 | 0.714 | 0.877 |
|  | Sh3a | 11 | 2 | 0.364 | 0.519 | 0.542 |
|  | Sh3o | 11 | 3 | 0.182 | 0.329 | 0.099 |
|  | Sh5c | 11 | 3 | 0.364 | 0.394 | 0.439 |
|  | Sh6e | 11 | 3 | 0.364 | 0.567 | 0.297 |
|  | Sh6L | 11 | 2 | 0.455 | 0.455 | 1.000 |
| Narawntapu NP 2014 | MHC101 | 17 | 2 | 0.706 | 0.499 | 0.137 |
|  | MHC102 | 17 | 2 | 0.765 | 0.508 | 0.053 |
|  | MHC105 | 15 | 2 | 0.667 | 0.515 | 0.325 |
|  | MHC106 | 16 | 3 | 0.500 | 0.522 | 1.000 |
|  | MHC107 | 17 | 7 | 0.706 | 0.774 | 0.187 |
|  | MHC108 | 17 | 4 | 0.706 | 0.661 | 1.000 |
|  | MHC109 | 17 | 2 | 0.824 | 0.499 | 0.010* |
|  | MHC110 | 17 | 2 | 0.353 | 0.471 | 0.338 |
|  | MHC111 | 17 | 4 | 0.471 | 0.544 | 0.117 |
|  | MHC202 | 17 | 4 | 0.529 | 0.506 | 1.000 |
|  | MHC203 | 17 | 2 | 0.176 | 0.166 | 1.000 |
|  | Sh2b | 16 | 2 | 0.313 | 0.353 | 1.000 |
|  | Sh2g | 17 | 3 | 0.412 | 0.355 | 1.000 |
|  | Sh2i | 17 | 3 | 0.588 | 0.661 | 0.450 |
|  | Sh2L | 17 | 2 | 0.294 | 0.258 | 1.000 |
|  | Sh2p | 17 | 2 | 0.412 | 0.337 | 1.000 |
|  | Sh2v | 17 | 4 | 0.647 | 0.597 | 0.119 |
|  | Sh3a | 17 | 2 | 0.588 | 0.428 | 0.242 |
|  | Sh3o | 17 | 2 | 0.176 | 0.337 | 0.095 |
|  | Sh5c | 17 | 3 | 0.647 | 0.528 | 0.816 |
|  | Sh6e | 17 | 2 | 0.529 | 0.401 | 0.276 |
|  | Sh6L | 17 | 2 | 0.176 | 0.451 | 0.020* |
|  | Sha001 | 17 | 3 | 0.412 | 0.437 | 0.367 |
|  | Sha008 | 17 | 4 | 0.529 | 0.549 | 0.536 |
|  | Sha009 | 17 | 2 | 0.294 | 0.258 | 1.000 |
|  | Sha010 | 17 | 4 | 0.588 | 0.544 | 0.563 |
|  | Sha011 | 17 | 1 |  |  |  |
|  | Sha013 | 17 | 4 | 0.882 | 0.701 | 0.466 |
|  | Sha014 | 17 | 3 | 0.294 | 0.558 | 0.000* |
|  | Sha015 | 17 | 2 | 0.294 | 0.487 | 0.138 |
|  | Sha023 | 17 | 4 | 0.706 | 0.683 | 1.000 |
|  | Sha024 | 17 | 2 | 0.588 | 0.499 | 0.625 |
|  | Sha025 | 17 | 2 | 0.294 | 0.258 | 1.000 |
|  | Sha026 | 17 | 3 | 0.294 | 0.269 | 1.000 |
|  | Sha028 | 15 | 3 | 0.400 | 0.453 | 0.295 |
|  | Sha033 | 17 | 2 | 0.059 | 0.059 | 1.000 |
|  | Sha034 | 17 | 2 | 0.412 | 0.401 | 1.000 |
|  | Sha036 | 17 | 2 | 0.529 | 0.401 | 0.276 |
|  | Sha037 | 17 | 4 | 0.882 | 0.672 | 0.195 |
|  | Sha039 | 17 | 3 | 0.824 | 0.622 | 0.300 |
|  | Sha040 | 17 | 4 | 0.706 | 0.690 | 0.438 |
|  | Sha042 | 17 | 2 | 0.471 | 0.428 | 1.000 |
| Stony Head | MHC101 | 24 | 2 | 0.500 | 0.454 | 1.000 |
|  | MHC102 | 24 | 2 | 0.500 | 0.454 | 1.000 |
|  | MHC105 | 23 | 2 | 0.261 | 0.294 | 0.520 |
|  | MHC106 | 24 | 2 | 0.542 | 0.510 | 1.000 |
|  | MHC107 | 24 | 5 | 0.792 | 0.745 | 0.771 |
|  | MHC108 | 24 | 4 | 0.875 | 0.685 | 0.140 |
|  | MHC109 | 24 | 2 | 0.417 | 0.496 | 0.672 |
|  | MHC110 | 24 | 3 | 0.750 | 0.621 | 0.678 |
|  | MHC111 | 24 | 4 | 0.458 | 0.568 | 0.017* |
|  | MHC202 | 24 | 4 | 0.583 | 0.719 | 0.262 |
|  | MHC203 | 18 | 3 | 0.333 | 0.298 | 1.000 |
|  | Sh2b | 23 | 2 | 0.087 | 0.085 | 1.000 |
|  | Sh2g | 24 | 3 | 0.458 | 0.488 | 0.366 |
|  | Sh2i | 24 | 2 | 0.333 | 0.284 | 1.000 |
|  | Sh2L | 24 | 2 | 0.083 | 0.082 | 1.000 |
|  | Sh2p | 24 | 1 |  |  |  |
|  | Sh2v | 24 | 5 | 0.458 | 0.474 | 0.333 |
|  | Sh3a | 24 | 2 | 0.417 | 0.496 | 0.672 |
|  | Sh3o | 24 | 3 | 0.583 | 0.598 | 0.642 |
|  | Sh5c | 22 | 3 | 0.636 | 0.486 | 0.279 |
|  | Sh6e | 24 | 3 | 0.375 | 0.481 | 0.453 |
|  | Sh6L | 24 | 2 | 0.333 | 0.496 | 0.201 |
|  | Sha001 | 24 | 3 | 0.417 | 0.547 | 0.087 |
|  | Sha008 | 24 | 4 | 0.542 | 0.680 | 0.240 |
|  | Sha009 | 24 | 1 |  |  |  |
|  | Sha010 | 24 | 3 | 0.542 | 0.526 | 0.296 |
|  | Sha011 | 24 | 2 | 0.000 | 0.082 | 0.021* |
|  | Sha013 | 24 | 4 | 0.750 | 0.719 | 0.754 |
|  | Sha014 | 24 | 2 | 0.375 | 0.439 | 0.635 |
|  | Sha015 | 24 | 2 | 0.375 | 0.439 | 0.635 |
|  | Sha023 | 21 | 5 | 0.714 | 0.643 | 0.772 |
|  | Sha024 | 17 | 2 | 0.353 | 0.371 | 1.000 |
|  | Sha025 | 23 | 2 | 0.391 | 0.449 | 0.643 |
|  | Sha026 | 24 | 4 | 0.708 | 0.698 | 0.978 |
|  | Sha028 | 24 | 3 | 0.583 | 0.585 | 0.168 |
|  | Sha033 | 24 | 1 |  |  |  |
|  | Sha034 | 24 | 2 | 0.167 | 0.156 | 1.000 |
|  | Sha036 | 23 | 2 | 0.348 | 0.294 | 1.000 |
|  | Sha037 | 22 | 4 | 0.591 | 0.663 | 0.254 |
|  | Sha039 | 22 | 3 | 0.455 | 0.588 | 0.440 |
|  | Sha040 | 24 | 3 | 0.542 | 0.479 | 0.662 |
|  | Sha042 | 24 | 2 | 0.333 | 0.507 | 0.116 |
| wukalina / Mt. William NP | MHC101 | 19 | 2 | 0.474 | 0.508 | 1.000 |
|  | MHC102 | 19 | 2 | 0.421 | 0.478 | 0.646 |
|  | MHC105 | 18 | 2 | 0.278 | 0.500 | 0.138 |
|  | MHC106 | 19 | 3 | 0.526 | 0.512 | 1.000 |
|  | MHC107 | 19 | 7 | 0.684 | 0.750 | 0.806 |
|  | MHC108 | 19 | 4 | 0.632 | 0.649 | 0.670 |
|  | MHC109 | 19 | 3 | 0.368 | 0.531 | 0.157 |
|  | MHC110 | 19 | 3 | 0.526 | 0.551 | 1.000 |
|  | MHC111 | 19 | 4 | 0.632 | 0.559 | 0.886 |
|  | MHC202 | 19 | 5 | 0.684 | 0.667 | 0.383 |
|  | MHC203 | 19 | 4 | 0.211 | 0.619 | 0.000* |
|  | Sh2b | 19 | 2 | 0.105 | 0.102 | 1.000 |
|  | Sh2g | 19 | 2 | 0.474 | 0.462 | 1.000 |
|  | Sh2i | 19 | 2 | 0.053 | 0.053 | 1.000 |
|  | Sh2L | 19 | 2 | 0.158 | 0.149 | 1.000 |
|  | Sh2p | 19 | 2 | 0.053 | 0.053 | 1.000 |
|  | Sh2v | 19 | 5 | 0.474 | 0.651 | 0.104 |
|  | Sh3a | 19 | 2 | 0.474 | 0.371 | 0.525 |
|  | Sh3o | 19 | 4 | 0.368 | 0.579 | 0.023* |
|  | Sh5c | 19 | 3 | 0.211 | 0.198 | 1.000 |
|  | Sh6e | 19 | 2 | 0.421 | 0.444 | 1.000 |
|  | Sh6L | 19 | 2 | 0.526 | 0.512 | 1.000 |
|  | Sha001 | 19 | 3 | 0.579 | 0.512 | 0.652 |
|  | Sha008 | 19 | 2 | 0.632 | 0.444 | 0.110 |
|  | Sha009 | 19 | 1 |  |  |  |
|  | Sha010 | 19 | 5 | 0.474 | 0.560 | 0.332 |
|  | Sha011 | 19 | 1 |  |  |  |
|  | Sha013 | 19 | 5 | 0.789 | 0.782 | 0.807 |
|  | Sha014 | 19 | 3 | 0.579 | 0.536 | 0.800 |
|  | Sha015 | 19 | 2 | 0.526 | 0.512 | 1.000 |
|  | Sha023 | 19 | 3 | 0.368 | 0.542 | 0.130 |
|  | Sha024 | 19 | 2 | 0.211 | 0.193 | 1.000 |
|  | Sha025 | 19 | 3 | 0.579 | 0.511 | 0.780 |
|  | Sha026 | 19 | 3 | 0.579 | 0.496 | 0.290 |
|  | Sha028 | 18 | 4 | 0.667 | 0.641 | 0.448 |
|  | Sha033 | 19 | 1 |  |  |  |
|  | Sha034 | 19 | 2 | 0.316 | 0.273 | 1.000 |
|  | Sha036 | 19 | 2 | 0.316 | 0.398 | 0.549 |
|  | Sha037 | 19 | 5 | 0.579 | 0.752 | 0.370 |
|  | Sha039 | 19 | 3 | 0.526 | 0.548 | 0.051 |
|  | Sha040 | 19 | 4 | 0.632 | 0.738 | 0.332 |
|  | Sha042 | 19 | 2 | 0.421 | 0.512 | 0.645 |

Abbr: *N*, number of animals genotyped; *N_A_*, number of alleles; *H_O_*, observed heterozygosity; *H_E_*, expected heterozygosity (for sample size using Levene’s correction [Levene, 1949])

^1^ Hardy-Weinberg exact test (Arlequin); * indicates statistically significant deviation from Hardy-Weinberg equilibrium at α = 0.05; note that only two of these (Sha014 at Narawntapu NP 2014 and MHC203 at wukalina / Mt. William NP) remained significant after correction for multiple testing (sequential Bonferroni correction [Holm, 1979]).

**Table S4** Allele frequencies for each locus in each study population^1^.

| **Locus ID** | **Allele ID** | **Narawntapu NP 2004** | **Narawntapu NP 2014** | **Stony Head** | **wukalina / Mt. William NP** |
| --- | --- | --- | --- | --- | --- |
| Sh2b^2^ | 112 | - | 0.781 | 0.957 | 0.947 |
|  | 116 | - | 0.219 | 0.043 | 0.053 |
| Sh2g^2^ | 116 | 0.409 | 0.794 | 0.688 | 0.342 |
|  | 118 | 0.091 | 0.147 | 0.146 | NA |
|  | 120 | 0.409 | 0.059 | 0.167 | 0.658 |
|  | 122 | 0.091 | NA | NA | NA |
| Sh2i^2^ | 227 | 0.136 | 0.206 | NA | NA |
|  | 229 | 0.591 | 0.412 | 0.833 | 0.974 |
|  | 231 | 0.273 | 0.382 | 0.167 | 0.026 |
| Sh2L^2^ | 173 | 0.227 | 0.147 | 0.042 | NA |
|  | 175 | NA | NA | NA | 0.079 |
|  | 177 | 0.773 | 0.853 | 0.958 | 0.921 |
| Sh2p^2^ | 143 | 0.409 | 0.206 | NA | 0.026 |
|  | 145 | 0.455 | 0.794 | 1.000 | 0.974 |
|  | 147 | 0.091 | NA | NA | NA |
|  | 149 | 0.045 | NA | NA | NA |
| Sh2v^2^ | 187 | NA | NA | NA | 0.026 |
|  | 189 | 0.091 | 0.029 | NA | NA |
|  | 191 | 0.409 | 0.441 | 0.167 | 0.395 |
|  | 193 | 0.364 | 0.471 | 0.708 | 0.447 |
|  | 195 | 0.045 | 0.059 | 0.021 | 0.053 |
|  | 199 | 0.091 | NA | 0.042 | NA |
|  | 204 | NA | NA | 0.063 | NA |
|  | 205 | NA | NA | NA | 0.079 |
| Sh3a^2^ | 186 | NA | 0.294 | 0.583 | 0.763 |
|  | 187 | 0.455 | NA | NA | NA |
|  | 188 | NA | 0.706 | 0.417 | 0.237 |
|  | 189 | 0.545 | NA | NA | NA |
| Sh3o^2^ | 224 | 0.091 | NA | NA | 0.053 |
|  | 226 | 0.818 | 0.794 | 0.563 | 0.605 |
|  | 228 | 0.091 | 0.206 | 0.250 | 0.105 |
|  | 230 | NA | NA | 0.188 | 0.237 |
| Sh5c^2^ | 113 | 0.136 | 0.118 | 0.091 | 0.026 |
|  | 119 | 0.773 | 0.647 | 0.682 | 0.895 |
|  | 121 | 0.091 | 0.235 | 0.227 | 0.079 |
| Sh6e^2^ | 202 | 0.318 | 0.265 | 0.333 | 0.316 |
|  | 204 | 0.591 | 0.735 | 0.646 | 0.684 |
|  | 210 | 0.091 | NA | 0.021 | NA |
| Sh6L^2^ | 147 | 0.318 | 0.676 | 0.417 | 0.526 |
|  | 149 | 0.682 | 0.324 | 0.583 | 0.474 |
| Sha001^3^ | 224 | - | NA | NA | NA |
|  | 226 | - | 0.735 | 0.625 | 0.632 |
|  | 228 | - | 0.147 | 0.146 | NA |
|  | 230 | - | 0.118 | 0.229 | 0.316 |
|  | 232 | - | NA | NA | 0.053 |
| Sha008^3^ | 241 | - | 0.118 | 0.042 | NA |
|  | 243 | - | 0.176 | 0.438 | 0.316 |
|  | 245 | - | 0.647 | 0.313 | 0.684 |
|  | 247 | - | NA | 0.208 | NA |
|  | 251 | - | 0.059 | NA | NA |
| Sha009^3^ | 244 | - | 0.147 | NA | NA |
|  | 246 | - | 0.853 | 1.000 | 1.000 |
| Sha010^3^ | 324 | - | 0.147 | 0.229 | 0.053 |
|  | 330 | - | 0.647 | 0.646 | 0.605 |
|  | 332 | - | 0.176 | NA | 0.026 |
|  | 336 | - | NA | NA | 0.026 |
|  | 338 | - | 0.029 | 0.125 | 0.289 |
|  | 344 | - | NA | NA | NA |
| Sha011^3^ | 239 | - | NA | 0.042 | NA |
|  | 242 | - | 1.000 | 0.958 | 1.000 |
| Sha013^3^ | 292 | - | NA | NA | 0.158 |
|  | 296 | - | 0.353 | 0.167 | 0.237 |
|  | 298 | - | 0.029 | 0.146 | 0.079 |
|  | 300 | - | 0.265 | 0.271 | 0.342 |
|  | 304 | - | 0.353 | 0.417 | 0.184 |
| Sha015^3^ | 306 | - | 0.618 | 0.313 | 0.526 |
|  | 310 | - | 0.382 | 0.688 | 0.474 |
| Sha024^3^ | 187 | - | 0.412 | 0.235 | 0.105 |
|  | 189 | - | 0.588 | 0.765 | 0.895 |
| Sha025^3^ | 235 | - | NA | NA | 0.026 |
|  | 236 | - | 0.147 | 0.674 | 0.368 |
|  | 237 | - | 0.853 | 0.326 | 0.605 |
| Sha026^3^ | 269 | - | 0.059 | 0.104 | 0.342 |
|  | 271 | - | 0.088 | 0.417 | 0.026 |
|  | 273 | - | NA | 0.146 | NA |
|  | 277 | - | 0.853 | 0.333 | 0.632 |
| Sha028^3^ | 173 | - | 0.267 | 0.208 | 0.333 |
|  | 175 | - | 0.700 | 0.583 | 0.500 |
|  | 177 | - | NA | NA | 0.056 |
|  | 179 | - | 0.033 | 0.208 | 0.111 |
| Sha032^3^ | 270 | - | 0.412 | 0.071 | 0.316 |
|  | 272 | - | 0.382 | 0.500 | 0.605 |
|  | 274 | - | 0.088 | 0.024 | 0.079 |
|  | 276 | - | 0.118 | 0.333 | NA |
|  | 280 | - | NA | 0.071 | NA |
| Sha033^3^ | 323 | - | 0.029 | NA | NA |
|  | 325 | - | 0.971 | 1.000 | 1.000 |
| Sha034^3^ | 221 | - | 0.265 | 0.083 | 0.158 |
|  | 223 | - | 0.735 | 0.917 | 0.842 |
| Sha036^3^ | 316 | - | 0.735 | 0.826 | 0.737 |
|  | 318 | - | 0.265 | 0.174 | 0.263 |
| Sha037^3^ | 204 | - | 0.235 | 0.432 | 0.263 |
|  | 206 | - | NA | NA | 0.105 |
|  | 208 | - | 0.265 | 0.182 | 0.263 |
|  | 210 | - | 0.471 | 0.364 | 0.342 |
|  | 212 | - | 0.029 | 0.023 | 0.026 |
| Sha039^3^ | 169 | - | 0.353 | 0.409 | 0.184 |
|  | 171 | - | 0.500 | 0.500 | 0.632 |
|  | 173 | - | NA | 0.091 | NA |
|  | 177 | - | 0.147 | NA | 0.184 |
| Sha040^3^ | 177 | - | 0.265 | 0.292 | 0.395 |
|  | 179 | - | 0.088 | NA | 0.158 |
|  | 191 | - | 0.471 | 0.667 | 0.237 |
|  | 193 | - | 0.176 | NA | 0.211 |
|  | 195 | - | NA | 0.042 | NA |
| Sha042^3^ | 211 | - | 0.706 | 0.542 | 0.526 |
|  | 215 | - | 0.294 | 0.458 | 0.474 |
| Sha14^3^ | 346 | - | 0.618 | 0.313 | 0.526 |
|  | 350 | - | 0.147 | NA | 0.026 |
|  | 352 | - | 0.235 | 0.688 | 0.447 |
| MHC101^4^ | 267 | - | 0.412 | 0.667 | 0.447 |
|  | 269 | - | 0.588 | 0.333 | 0.553 |
| MHC102^4^ | 201 | - | 0.559 | 0.333 | 0.368 |
|  | 203 | - | 0.441 | 0.667 | 0.632 |
| MHC105^4^ | 130 | - | 0.533 | 0.174 | 0.417 |
|  | 132 | - | 0.467 | 0.826 | 0.583 |
| MHC106^4^ | 146 | - | NA | NA | 0.316 |
|  | 148 | - | 0.219 | 0.521 | 0.632 |
|  | 150 | - | 0.656 | 0.479 | 0.053 |
|  | 152 | - | 0.125 | NA | NA |
| MHC107^4^ | 157 | - | 0.147 | 0.125 | 0.105 |
|  | 159 | - | 0.088 | NA | NA |
|  | 165 | - | 0.059 | NA | 0.079 |
|  | 169 | - | 0.412 | 0.146 | 0.263 |
|  | 171 | - | 0.206 | 0.396 | 0.421 |
|  | 173 | - | NA | 0.063 | 0.053 |
|  | 175 | - | 0.029 | 0.271 | 0.053 |
|  | 177 | - | 0.059 | NA | 0.026 |
| MHC108^4^ | 206 | - | NA | 0.021 | NA |
|  | 216 | - | 0.324 | 0.375 | 0.500 |
|  | 218 | - | 0.029 | NA | 0.026 |
|  | 220 | - | 0.176 | 0.354 | 0.184 |
|  | 222 | - | 0.471 | 0.250 | 0.289 |
| MHC109^4^ | 172 | - | 0.588 | 0.417 | 0.553 |
|  | 174 | - | NA | NA | 0.026 |
|  | 176 | - | 0.412 | 0.583 | 0.421 |
| MHC110^4^ | 245 | - | 0.647 | 0.292 | 0.553 |
|  | 249 | - | NA | 0.188 | 0.053 |
|  | 257 | - | 0.353 | 0.521 | 0.395 |
| MHC111^4^ | 149 | - | 0.088 | 0.104 | 0.053 |
|  | 151 | - | 0.206 | 0.625 | 0.342 |
|  | 152 | - | 0.059 | 0.083 | NA |
|  | 153 | - | 0.647 | 0.188 | 0.579 |
|  | 155 | - | NA | NA | 0.026 |
| MHC202^4^ | 277 | - | 0.088 | 0.104 | 0.105 |
|  | 281 | - | 0.676 | 0.292 | 0.447 |
|  | 283 | - | 0.029 | 0.208 | 0.026 |
|  | 285 | - | 0.206 | 0.396 | 0.368 |
|  | 287 | - | NA | NA | 0.053 |
| MHC203^4^ | 162 | - | NA | NA | 0.368 |
|  | 164 | - | 0.912 | 0.833 | 0.500 |
|  | 170 | - | NA | 0.056 | 0.026 |
|  | 172 | - | 0.088 | 0.111 | 0.105 |

^1^ “NA” indicates alleles that were absent from a population, “dash” indicates loci that were not genotyped.

^2^ Locus initially characterised by Jones et al., 2003; allele size labels may differ from previously published values as a result of variation in lab methods, but are standardised across populations within the current study (see Methods).

^3^ Locus characterised by Gooley et al., 2017

^4^ Locus characterised by Cheng and Belov, 2012

**Table S5** Observations of sex ratio and breeding rate amongst females trapped in June or July at Stony Head, which was supplemented in August 2016.

| Year | *N* (Males,  Females) | Breeding females (proportion) | Mean PY per female (SD) | Mean PY per breeder (SD) |
| --- | --- | --- | --- | --- |
| 2014 | 9 (4, 5) | 4 (0.800) | 2.80 (1.64) | 3.50 (0.58) |
| 2015 | 15 (8, 7) | 4 (0.571) | 2.00 (2.00) | 3.50 (1.00) |
| 2016 | 10 (5, 5) | 3 (0.600) | 2.00 (2.00) | 3.33 (1.15) |
| 2017 | 30 (14, 16) | 13 (0.813) | 2.69 (1.58) | 3.31 (0.95) |

Abbr: *N* = number trapped, PY = pouch young, SD = standard deviation


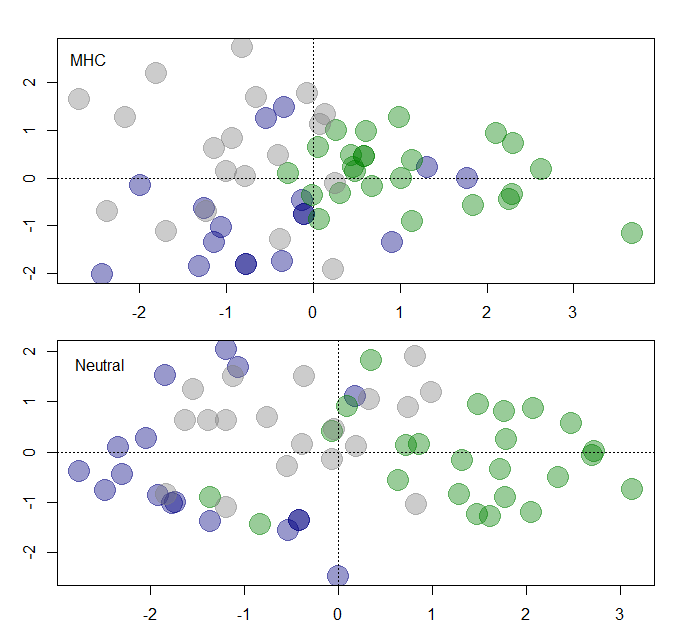
**Fig. S1** First two axes of DAPC of three populations of Tasmanian devils (blue = Narawntapu NP, grey = Stony Head, green = wukalina / Mt. William NP) based on either 11 MHC-linked microsatellite loci (“MHC”) or 31 putatively neutral microsatellite loci (“Neutral”). A plot based on a combined dataset is provided at Fig. 2


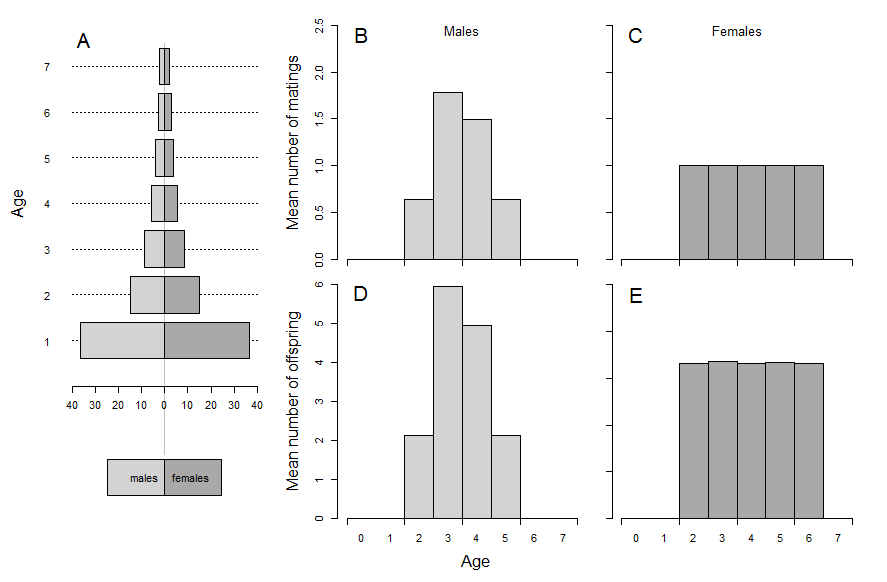


**Fig. S2** Population demography of the simulated Narawntapu NP population. Parameters shown are age structure of the population in year 50 (A), and the mean number of matings for males (B) and females (C) and mean number of offspring for males (D) and females (E) at each age. The latter four statistics are calculated as an average over all years of the simulation.


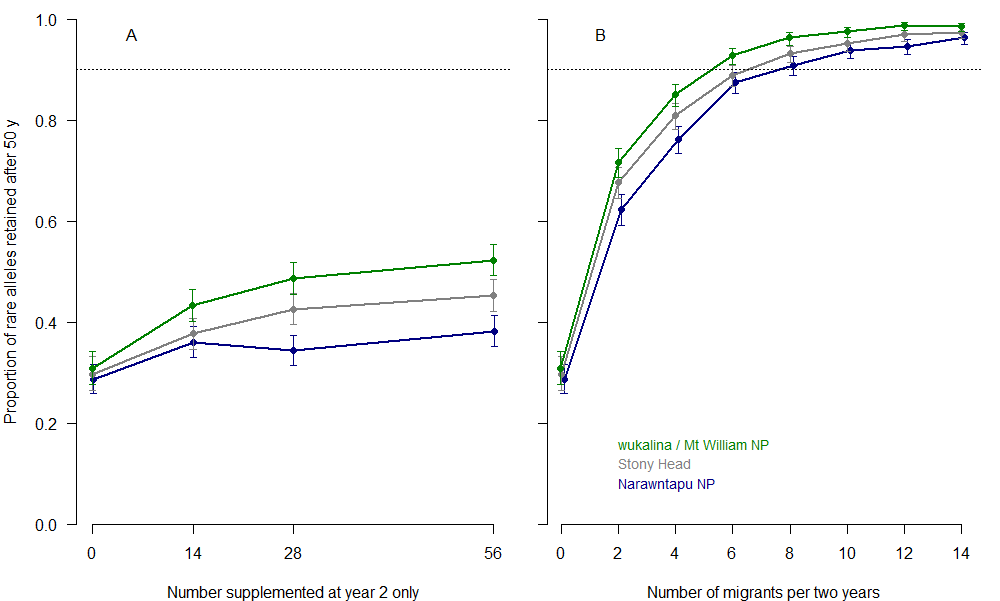


**Fig. S3** Populations in which migrants are given priority to breed (c.f. Fig. 5): effect of an initial, one-off supplementation (A) or two-yearly ongoing supplementation (B), at varying rates, on the retention of genetic diversity in three wild devil populations (as shown in legend). Each data point is the proportion of rare alleles retained after 50 years; error bars are the 95% confidence limit based on 1,000 replicates. The dashed line indicates the 90% retention goal. (Note that, for clarity, data points have been “offset” slightly with respect to the x-axis). Note that migrants are considered to come from a population with the same focal allele frequency (q = 0.05) as the starting condition of the recipient population.


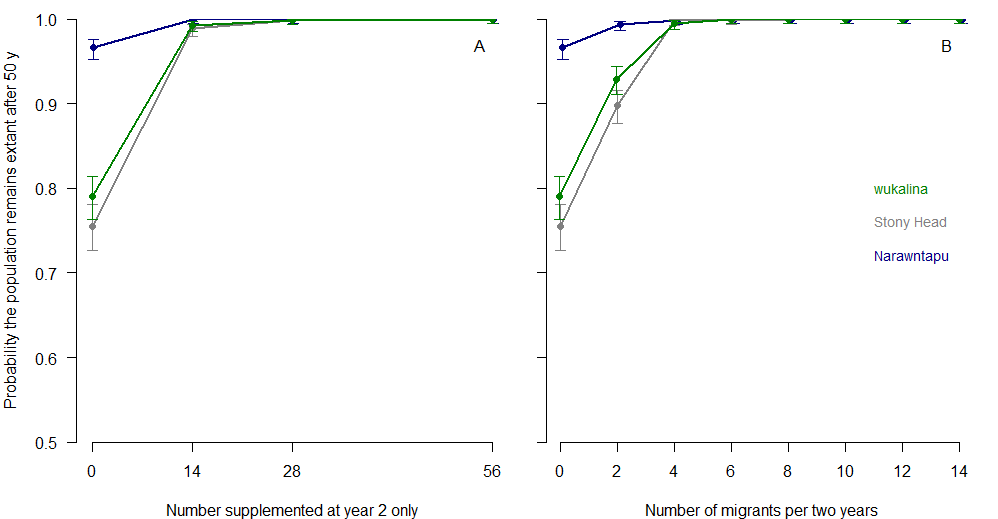


**Fig. S4** Effect of an initial, one-off supplementation (A) or two-yearly ongoing supplementation (B), at varying rates, on the probability of population persistence (proportion of 1,000 simulations in which the population did not go extinct). Error bars are the 95% confidence limits, note that the x-axis starts at 0.5.

**Literature cited in Supporting Information**

Cheng, Y., Belov, K. (2012) Isolation and characterisation of 11 MHC-linked microsatellite loci in the Tasmanian devil (*Sarcophilus harrisii*). Conservation Genetics Resources, 4, 463-465.

Gooley, R., Hogg, C.J., Belov, K., Grueber, C.E. (2017) No evidence of inbreeding depression in a Tasmanian devil insurance population despite significant variation in inbreeding. Scientific Reports, 7, 1830.

Grueber, C.E., Fox, S., Belov, K., Pemberton, D., Hogg, C.J. (2018) Landscape level field data reveals broad-scale effects of a fatal, transmissible cancer on population ecology of Tasmanian devil. Mammalian Biology - Zeitschrift für Säugetierkunde, 91, 41-45.

Hawkins, C., Baars, C., Hesterman, H., Hocking, G., Jones, M. (2006) Emerging disease and population decline of an island endemic, the Tasmanian devil *Sarcophilus harrisii*. Biological Conservation, 131, 307-324.

Holm, S. (1979) A simple sequentially rejective multiple test procedure. The Scandanavian Journal of Statistics, 6, 65-70.

Huxtable, S., Lee, A.V., Wise, P. (2015) Metapopulation management of an extreme disease scenario. In Advances in Reintroduction Biology of Australian and New Zealand Fauna, D Armstrong; MW Hayward; D Moro and PJ Seddon (editors). Clayton South, Victoria: CSIRO Publishing.

Jones, M.E., Paetkau, D., Geffen, E., Moritz, C. (2003) Microsatellites for the Tasmanian devil (*Sarcophilus laniarius*). Molecular Ecology Notes, 3, 277-279.

Jones, M.E., Paetkau, D., Geffen, E.L.I., Moritz, C. (2004) Genetic diversity and population structure of Tasmanian devils, the largest marsupial carnivore. Molecular Ecology, 13, 2197-2209.

Lachish, S., McCallum, H., Jones, M. (2009) Demography, disease and the devil: life-history changes in a disease-affected population of Tasmanian devils (*Sarcophilus harrisii*). Journal of Animal Ecology, 78, 427-436.

Lachish, S., Miller, K.J., Storfer, A., Goldizen, A.W., Jones, M.E. (2011) Evidence that disease-induced population decline changes genetic structure and alters dispersal patterns in the Tasmanian devil. Heredity, 106, 172-182.

Lazenby, B.T., Tobler, M.W., Brown, W.E., Hawkins, C.E., Hocking, G.J., Hume, F., Huxtable, S., Iles, P., Jones, M.E., Lawrence, C., Thalmann, S., Wise, P., Williams, H., Fox, S., Pemberton, D. (2018) Density trends and demographic signals uncover the long-term impact of transmissible cancer in Tasmanian devils. Journal of Applied Ecology, 55, 1368-1379.

Levene, H. (1949) On a matching problem arising in genetics. The Annals of Mathematical Statistics, 20, 91-94.

Miller, W., Hayes, V.M., Ratan, A., Petersen, D.C., Wittekindt, N.E., Miller, J., Walenz, B., Knight, J., Qi, J., Zhao, F., Wang, Q., Bedoya-Reina, O.C., Katiyar, N., Tomsho, L.P., Kasson, L.M., Hardie, R.-A., Woodbridge, P., Tindall, E.A., Bertelsen, M.F., Dixon, D., Pyecroft, S., Helgen, K.M., Lesk, A.M., Pringle, T.H., Patterson, N., Zhang, Y., Kreiss, A., Woods, G.M., Jones, M.E., Schuster, S.C. (2011) Genetic diversity and population structure of the endangered marsupial *Sarcophilus harrisii* (Tasmanian devil). Proceedings of the National Academy of Sciences, 108, 12348-12353.
